# Oxylipins mediate cell-to-cell communication in *Pseudomonas aeruginosa*

**DOI:** 10.1038/s42003-019-0310-0

**Published:** 2019-02-15

**Authors:** Eriel Martínez, Rachael K. Cosnahan, Mousheng Wu, Shiva. K. Gadila, Eric B. Quick, James A. Mobley, Javier Campos-Gómez

**Affiliations:** 1 Department of Infectious Diseases, Drug Discovery Division, Southern Research, Birmingham, AL USA; 2 Chemistry Department, Drug Discovery Division, Southern Research, Birmingham, AL USA; 3 Mass Spectrometry/Proteomics (MSP) Shared Facility, School of Medicine, University of Alabama at Birmingham, Birmingham, AL USA

## Abstract

Oxygenated unsaturated fatty acids, known as oxylipins, are signaling molecules commonly used for cell-to-cell communication in eukaryotes. However, a role for oxylipins in mediating communication in prokaryotes has not previously been described. Bacteria mainly communicate via quorum sensing, which involves the production and detection of diverse small molecules termed autoinducers. Here we show that oleic acid-derived oxylipins produced by *Pseudomonas aeruginosa* function as autoinducers of a novel quorum sensing system. We found that this system controls the cell density-dependent expression of a gene subset independently of the quorum sensing systems thus far described in this bacterium. We identified a LysR-type transcriptional regulator as the primary receptor of the oxylipin signal. The discovery of this oxylipin-dependent quorum sensing system reveals that prokaryote-derived oxylipins also mediate cell-to-cell communication in bacteria.

## Abstract (web summary)

Eriel Martínez et al. report that the bacterial pathogen *Pseudomonas aeruginosa* can convert oleic acids into oxylipins for use in cell-cell communication. This quorum sensing system is regulated by the bacterial protein called oxylipin-dependent diol synthase regulator OdsR.

## Introduction

Bacteria regulate gene expression in response to changes in cell density using a sophisticated cell-to-cell communication process known as quorum sensing. Quorum sensing controls biochemical pathways that are not needed in an isolated individual cell, but become beneficial as part of a population^1^. Diverse quorum sensing systems regulate important biological processes such as bioluminescence, DNA transfer, antibiotic resistance, motility, biofilm formation and virulence^2^. Population density is perceived through the synthesis, release and detection by the bacterial cells of small diffusible molecules referred to as autoinducers^3^. An increase in the bacterial population causes a proportional increase in the extracellular concentration of the autoinducers^4^. Once a threshold concentration is reached, they are detected by quorum sensing signal receptors that trigger a high cell density-specific gene expression program^5^.

In Gram-negative bacteria, *N*-acyl homoserine lactones (AHLs) are the most common class of autoinducers^6^. However, several bacterial species (Gram-positive and -negative) possess alternative quorum sensing mediators, such as alkylquinolones, α-hydroxyketones, peptides and fatty acid-like molecules^4^. The Gram-negative pathogen *Pseudomonas aeruginosa* offers one of the best studied models of quorum sensing networks in bacteria. Four interconnected quorum sensing systems have been described in this bacterium thus far: *las*, *rhl*, PQS (*Pseudomonas* quinolone signal) and IQS (Integrating quorum signal)^7–10^. The quorum sensing system *las* uses 3-oxo-C12-homoserine lactone, while *rhl* uses *N*-butyrylhomoserine lactone (both AHLs) as autoinducers. PQS and IQS on the other hand produce and sense the mediators 3,4-dihydroxy-2-heptylquinoline and 2-(2-hydroxyphenyl)-thiazole-4-carbaldehyde, respectively^11^. These quorum sensing systems establish diverse interactions among them, creating a complex regulatory quorum sensing circuitry^11^.

Oxylipins are common cell-to-cell communication mediators in eukaryotes^12^. However, very little is known about the role of prokaryote-derived oxylipins in bacterial physiology. *P. aeruginosa* possesses a fatty acid diol synthase activity that catalyzes the stereospecific oxygenation of exogenous oleic acid (OA)^13^. The enzymes responsible for this activity are two fatty acid-di-heme cytochrome *C* peroxidases localized in the periplasm^14,15^. We recently reported that the oxylipins (10*S*)-hydroxy-(8*E*)-octadecenoic acid (10-HOME) and 7*S*,10*S*-dihydroxy-(8*E*)-octadecenoic acid (7,10-DiHOME) generated by the diol synthase activity are involved in several biological processes, such as motility, biofilm formation and virulence in *P. aeruginosa*^16^. Compelled by these findings and the role of eukaryote-derived oxylipins in cell-to-cell communication, we investigated whether diol synthase-derived oxylipins are involved in cell-to-cell communication in *P. aeruginosa*. We found that *P. aeruginosa* produces and senses oxylipins in a cell density-dependent manner through a novel quorum sensing system we termed ODS (*o*xylipin-*d*ependent quorum sensing *s*ystem). This system operates independently of the hierarchical quorum sensing circuitry of *P. aeruginosa*. We identified the protein encoded by *PA2076* gene of PAO1 as the primary receptor of oxylipins in this bacterium. This protein, which we refer to as OdsR (*o*xylipin-dependent *d*iol *s*ynthase *r*egulator), is a LysR-type transcriptional regulator (LTTR) that mediates oxylipin-dependent induction of the diol synthase enzymes. The discovery of ODS reveals for the first time that prokaryote-derived oxylipins are also signaling molecules mediating cell-to-cell communication in bacteria.

## Results

### A positive regulatory loop controls the diol synthase operon expression

**Fig. 1:**
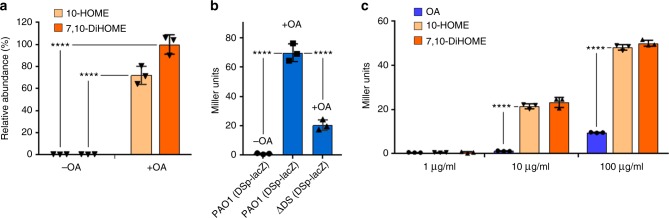
10-HOME and 7,10-DiHOME induce the expression of the diol synthase operon. **a** In vitro bioconversion of oleic acid (OA) into 10-HOME and 7,10-DiHOME oxylipins using the periplasmic fraction isolated from PAO1. The expression of the diol synthase enzymes was dependent on culturing PAO1 in the presence of OA. **b** Expression of β-galactosidase (β-gal) activity in PAO1 (pDSp*-lacZ*) and ΔDS (pDSp*-lacZ*) treated with OA (1 mg mL^−1^). No β-gal activity was detected in PAO1 (pDSp*-lacZ*) in the absence of OA. OA induced at least threefold more β-gal activity in PAO1 (pDSp*-lacZ*) than ΔDS (pDSp*-lacZ*). **c** Expression of β-gal activity in ΔDS (DSp*-lacZ*) treated with OA or oxylipins at the shown concentrations. ΔDS (pDSp*-lacZ*) showed fivefold more β-gal activity when treated with 10-HOME or 7,10-DiHOME than with OA and the increase in β-gal expression was dose dependent. Means and s.d. are from three independent experiments. 10-HOME (10*S*)-hydroxy-(8*E*)-octadecenoic acid, 7,10-DiHOME 7*S*,10*S*-dihydroxy-(8*E*)-octadecenoic acid. ****Significantly different, unpaired two-tailed *t*-test, *P* < 0.0001

The diol synthase enzymes of *P. aeruginosa* are encoded by the *PA2077* and *PA2078* genes, which together form an operon (Supplementary Fig. 1)^14^. Once expressed, these enzymes localize mainly in the periplasm^15^. We found that addition of OA to the culture was required to isolate a periplasmic fraction of *P. aeruginosa* displaying diol synthase activity in vitro (Fig. 1a). This observation suggested that expression of the diol synthase enzymes is dependent on exogenous OA. To confirm this we used *P. aeruginosa* PAO1 strain containing a genetic fusion between the diol synthase promoter and the *Escherichia coli lacZ* reporter gene cloned into plasmid pDSp*-lacZ* (Supplementary Table 1). The β-galactosidase (β-gal) activity in this strain was dependent on the addition of OA to the medium (Fig. 1b). Surprisingly, when pDSp*-lacZ* was introduced in a diol synthase-lacking background strain, ΔDS (pDSp*-lacZ*), the OA failed to fully induce the expression of β-gal activity (Fig. 1b, third column). This result indicated that oxylipins derived from the diol synthase activity on OA were required to fully induce expression of the diol synthase operon. We next tested the ability of 10-HOME and 7,10-DiHOME oxylipins, purified from a PAO1 culture supernatant, to induce the β-gal activity in ΔDS (pDSp*-lacZ*). Each oxylipin, used at 0.1 mg mL^−1^, induced the β-gal activity in ΔDS (pDSp*-lacZ*) more than fivefold greater than OA used at the same concentration (Fig. 1c). Altogether, these data indicate that production of oxylipins in *P. aeruginosa* is regulated by a positive regulatory circuit in which oxylipins induce full expression of their own biosynthetic enzymes.

### Oxylipin production depends on cell density

**Fig. 2:**
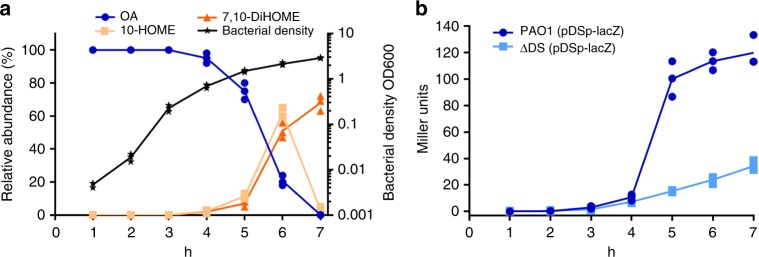
Cell density-dependent expression of the diol synthase operon. **a** Time course of oxylipin production during the culture growth. Oxylipins started to be produced at the late exponential phase (4 h, OD~0.6). Then, the amount of oxylipins increased around 50 times in approximately 2 h. **b** Time course of β-galactosidase (β-gal) activity in PAO1 (pDSp-*lacZ*) and ΔDS (pDSp-*lacZ*) grown in the presence of 1 mg mL^−1^ of oleic acid (OA). The diol synthase operon was poorly induced in ΔDS (pDSp-*lacZ*). Means and s.d. are from three independent experiments

Positive autoregulatory circuits, in which the autoinducer molecules positively regulate their own synthesis in a cell density-dependent manner, are characteristic of quorum sensing systems^17,18^. Based on these data we reasoned that oxylipin accumulation in the supernatant of PAO1 could follow a production kinetics similar to that of quorum sensing autoinducers. To test this, we performed a kinetic experiment to follow the accumulation of oxylipins in the supernatant of PAO1 cultured in M63 medium supplemented with 1 mg mL^−1^ of OA. No detectable amounts of oxylipins were found in the first 3 h of growth (exponential phase), whereas the concentration of oxylipins rapidly increased after 4 h of growth (early stationary phase) (Fig. 2a). In addition, after 6 h, when the OA was mostly consumed, the concentration of 10-HOME rapidly decreased, while the 7,10-DiHOME oxylipin remained longer in the culture. This experiment suggested that production of 10-HOME and 7,10-DiHOME oxylipins is regulated in a cell density-dependent manner, although 7,10-DiHOME had a slower kinetics of consumption compared to 10-HOME (Fig. 2a). To determine the level of the diol synthase operon expression in relation to the cell density, the kinetics of β-gal activity in PAO1 (pDSp*-lacZ*) was monitored. The expression of β-gal activity was evident at the early stationary phase of the culture, rapidly increasing (from 4 to 5 h), following a sigmoid curve (Fig. 2b, in dark blue). When the same experiment was done with ΔDS (pDSp*-lacZ*), there was poor expression of the diol synthase operon (Fig. 2b, in pale blue). These results suggest that expression of the diol synthase operon is regulated in a cell density-dependent manner through a positively autoregulated circuit mediated by 10-HOME and 7,10-DiHOME.

### PA2076 protein controls the expression of the diol synthase operon

**Fig. 3:**
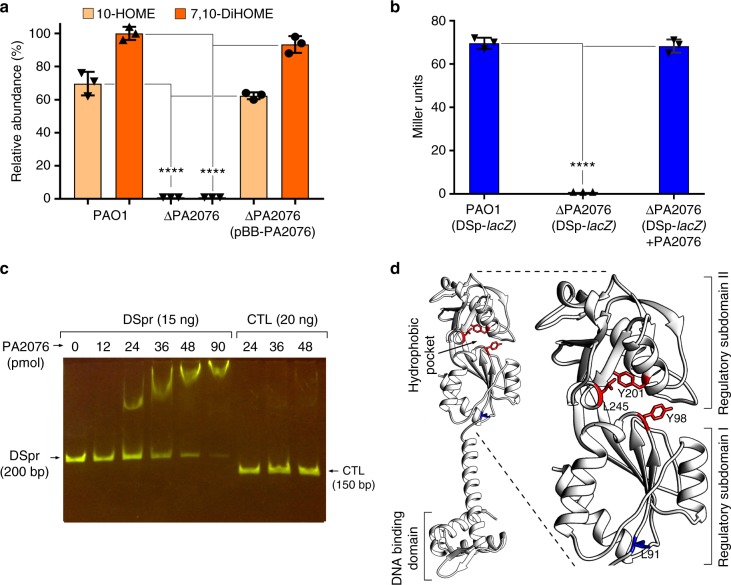
OdsR (oxylipin-dependent diol synthase regulator) is an oxylipin-dependent inducer of the diol synthase operon. **a** Production of oxylipins in the supernatant of PAO1, Δ*PA2076* and Δ*PA2076* complemented with a copy of *odsR* gene (Δ*PA2076*/pBB-*odsR*-His). Δ*PA2076* failed to produce oxylipins. **b** β-Galactosidase (β-gal) expression in PAO1 (pDSp-*lacZ*), Δ*PA2076* (pDSp-*lacZ*) and Δ*PA2076* complemented with *PA2076* [Δ*PA2076* (pDSp-*lacZ*+pBB-*odsR*-His)]. Δ*PA2076* (pDSp-*lacZ*) did not express detectable β-gal activity in the presence of oleic acid (OA; 1 mg mL^−1^) (*t*-test, *P* < 0.0001). **c** Electrophoretic mobility shift assay (EMSA) gel showing specific binding of OdsR protein to the diol synthase promoter probe (DSpr, 200 bp), but not to a control unrelated DNA probe (CTL, 150 bp). **d** I-TASSER predicted model of OdsR three-dimensional (3D) structure. The hydrophobic pocket localized in between the two regulatory subdomains is indicated. The hydrophobic amino acids in the pocket that were replaced by hydrophilic counterparts are represented in red. These changes abolished the ability of OdsR to induce the β-gal activity of the DS-*lacZ* genetic fusion. The amino acid L91 (in blue) outside of the pocket had no effect on OdsR function. Data from **a**, **b** are the means and s.d. of three independent experiments. ****Significantly different, unpaired two-tailed *t*-test, *P* < 0.0001

A positive autoregulatory circuit usually involves a transcriptional regulator that binds the inducer molecule and in turn activates the expression of the inducer biosynthetic enzymes^19^. In many cases, the transcriptional regulator-encoding gene localizes near the genes encoding the autoinducer biosynthetic enzymes (e.g., LasR–LasI and RhlR–RhlI pairs in *P. aeruginosa or* LuxR-LuxI *in Vibrio fischeri*). In PAO1, the gene *PA2076*, encoding a putative LTTR, localizes downstream of the diol synthase operon (Supplementary Fig. 1A). In order to determine if the product of *PA2076* was involved in regulation of the diol synthase operon, a *PA2076* deletion mutant was created (Δ*PA2076*). It was found that Δ*PA2076* failed to produce oxylipins in M63 supplemented with OA (Fig. 3a). To rule out any polar effect potentially caused by *PA2076* deletion on the contiguous diol synthase operon, *PA2076* protein was expressed in trans from plasmid pBB-*odsR-His*. The ability of the complemented Δ*PA2076* strain to produce oxylipin recovered (Fig. 3a). In addition, it was shown that Δ*PA2076* (pDSp*-lacZ*) did not express detectable amounts of β-gal activity, indicating that *PA2076* is required for expression of the diol synthase operon (Fig. 3b). The β-gal activity in Δ*PA2076* (pDSp*-lacZ*) was restored by reintroducing *PA2076* into the chromosome. These results suggest that *PA2076* encodes a positive transcriptional regulator (hereafter termed OdsR) of the operon. An electrophoretic mobility shift assay (EMSA) using a purified His-tag version of OdsR (OdsR-His) showed that OdsR specifically bound to a DNA probe consisting of the 200 bp sequence immediately upstream of the first gene (*PA2078*) of the diol synthase operon, but not to an unrelated DNA probe used as control (Fig. 3c, Supplementary Fig. 2).

Some transcriptional regulators involved in positive autoregulatory feedback are able to induce their own expression in the presence of their respective autoinducers (e.g., LasR and RhlR). To assess whether OdsR was induced by OA or its derivative oxylipins, a His-tag version of the *odsR* gene was cloned in the pBBR1MCS vector under control of its native promoter, yielding the plasmid pBB-*odsR*-His (Supplementary Table 1). A western blot analysis using an anti-poly-histidine antibody indicated that PAO1 (pBB-*odsR*-His) produced OdsR-His at the same level in the presence or absence of OA. This suggests that OdsR is constitutively expressed in *P. aeruginosa* (Supplementary Fig. 1,B). The EMSA experiment also showed that OdsR binds to the diol synthase operon promoter in the presence or absence of the oxylipins. These results suggest that upon binding to the OdsR-DNA complex, the oxylipins induce a conformational change that allows the RNA polymerase to transcribe the diol synthase operon. This is similar to the mechanism of action of other known LTTRs^20^.

LTTRs contain a DNA binding domain and a regulatory domain formed by two subdomains. The inducer molecule usually binds to the region between the two subdomains, inducing a conformational change of the protein that activates gene expression^20^. We used the online I-TASSER server to predict the three-dimensional (3D) structure of OdsR. It predicted a large hydrophobic pocket in the inter-subdomain region of the regulatory domain that might serve as the binding site for the oxylipins (Fig. 3d). In agreement with this hypothesis, it was determined that three independent mutations (Y98Q, L143Q and L245T), in which three non-adjacent hydrophobic amino acids in this region were replaced by hydrophilic amino acids, abolished the oxylipin-dependent induction of the diol synthase activity in PAO1 (Supplementary Fig. 1C). As a control, mutagenesis of an amino acid localized outside of the predicted hydrophobic pocket had no effect on the diol synthase activity (Supplementary Fig. 1C). These results suggest that the hydrophobic pocket found in the predicted structure of OdsR is an oxylipin binding site.

### Oxylipins control a gene subset expression

Previously it was found that 10-HOME and 7,10-DiHOME oxylipins play a role in several physiological processes in *P. aeruginosa*^16^. However, the specific metabolic pathways regulated by oxylipins in *P. aeruginosa* remain unknown. To identify proteins specifically induced by oxylipins, a comparative proteomic analysis of PAO1 and Δ*odsR*, both grown in the presence and absence of OA or each of the oxylipins, was performed. In general, we identified 17 proteins that were induced (excluding PA2077 and PA2078) and 16 proteins that were inhibited more than fivefold by 10-HOME and/or 7,10-DiHOME in the proteomic analysis (Supplementary Table 2). As expected, oxylipin-dependent induction of the diol synthase enzymes (PA2077 and PA2078) occurred in PAO1, but not in Δ*odsR*. In fact, no other protein, apart from PA2077 and PA2078, seems to be directly regulated by OdsR. This observation suggests that OdsR is restricted to regulating the diol synthase operon and therefore controls the amount of oxylipins produced in a cell density-dependent manner. However, a set of proteins were up- or down-shifted fivefold or more in both PAO1 and Δ*odsR* when treated with oxylipins. These findings suggest that the oxylipins regulate the expression of these proteins through mediation of other as yet unidentified transcriptional regulators that use 10-HOME or 7,10-DiHOME oxylipins as inducers.

We also analyzed the transcriptome of Δ*odsR* in the presence or absence of OA or each of the oxylipins. We identified 29 genes that were induced by oxylipins and 10 genes that were repressed fivefold or more (Supplementary Table 3). As expected, most of the proteins identified as regulated by oxylipins were identified in both the proteomics and the transcriptomic studies (Supplementary Tables 2 and 3). In addition, we identified differences in the proteome and the transcriptome analysis that might reflect the sensitivity of each method and/or the specificities in posttranscriptional regulation. In both studies we found a stronger effect of 10-HOME than that observed for 7,10-DiHOME.

To confirm the validity of the -omics studies, *lacZ* was transcriptionally fused with the promoter of *PA3427* as a representative gene of those strongly induced by the oxylipins. PAO1 strain containing this fusion [PAO1 (*PA3427*p-*lacZ*] expressed β-gal activity only when grown in the presence of OA (Supplementary Fig. 3). However, Δ*odsR* (*PA3427*p-*lacZ*) grown in OA displayed very poor β-gal activity. This observation validated the findings of the proteome and the transcriptome studies by confirming that at least the *PA3427* gene is up-regulated by the oxylipins derived from the diol synthase activity. It was also shown that purified 10-HOME and 7,10-DiHOME induced β-gal activity in both strains, PAO1 (*PA3427*p-*lacZ*) and Δ*odsR* (*PA3427*p-*lacZ*) (Supplementary Fig. 3), indicating that *PA3427* is regulated by oxylipins independently of OdsR and through an, as of yet, unidentified transcriptional regulator.

### Oxylipins function as quorum sensing signals

**Fig. 4:**
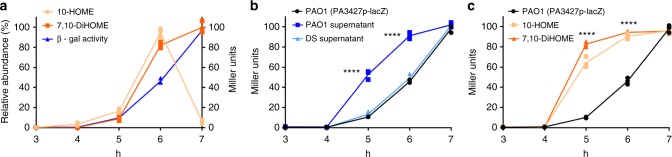
The oxylipin-dependent quorum sensing system (ODS) regulates cell density-dependent expression of *PA3427*. **a** Time course of β-galactosidase (β-gal) activity in PAO1 (*PA3427*p*-lacZ*) during culture growth. The β-gal activity is expressed in a cell density-dependent manner which correlated with the kinetics of oxylipin production. **b** The β-gal activity is prematurely expressed at earlier time points of growth when treated with free-cell supernatants of PAO1 stationary phase cultures (oxylipin rich supernatants) or **c** with purified 10-HOME or 7,10-DiHOME. Means and s.d. are from three independent experiments. 10-HOME (10*S*)-hydroxy-(8*E*)-octadecenoic acid, 7,10-DiHOME 7*S*,10*S*-dihydroxy-(8*E*)-octadecenoic acid. ****Significantly different to control [PAO1 (*PA3427*p-lacZ)] at time points 4 and 5 h, per time point unpaired two-tailed *t*-test, *P* < 0.0001

The observation that *P. aeruginosa* produces 10-HOME and 7,10-DiHOME oxylipins in a cell density-dependent manner suggests that those genes outside of the diol synthase operon and regulated by oxylipins should also follow a cell density-dependent expression pattern. To evaluate this hypothesis, an expression kinetics assay was performed by following the β-gal activity in PAO1 (*PA3427*p-*lacZ*). This strain expressed β-gal activity in a cell density-dependent manner, correlating with the level of oxylipins in the culture supernatant (Fig. 4a). This effect was similar to that observed with quorum sensing-regulated genes. For example, *PA3427*p-*lacZ* was prematurely induced when free-cell supernatants of stationary cultures of PAO1 (containing high level of oxylipins) were added to fresh cultures at 4 h (Fig. 4b). As expected, this was not observed when free-cell supernatants of ΔDS cultures at stationary phase were added (Fig. 4b). In addition, pure oxylipins, 10-HOME or 7,10-DiHOME, prematurely induced β-gal activity when added to PAO1 (*PA3427*p-l*acZ*) cultures at early exponential phase (Fig. 4c). Altogether, these results demonstrate that oxylipins produced by *P. aeruginosa* are autoinducers of an oxylipin-dependent quorum sensing system that we refer to as ODS.

**Fig. 5:**
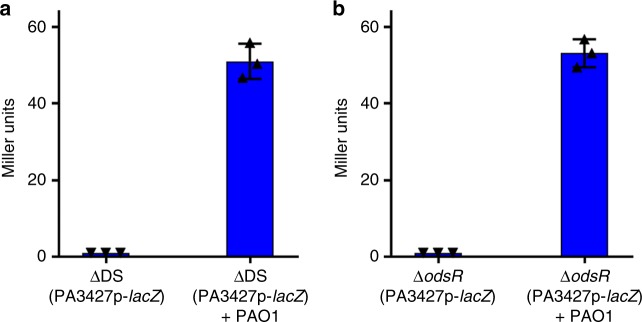
*Oxylipin-deficient P. aeruginosa* profit from oxylipins produced by PAO1 wild-type strain. **a** Graph showing the β-galactosidase (β-gal) activity of ΔDS (*PA3427*p-*lacZ*) grown alone or in co-culture with PAO1. **b** Graph of the same experiment, but using Δ*odsR* (*PA3427*p-*lacZ*)

The quorum sensing signal molecules are “social public goods” that can be exploited by signal-producing deficient mutants. For example, a *lasI* mutant of *P. aeruginosa* unable to produce 3O-C12-HSL responds to the signal produced by a wild-type strain in co-culture^21^. This process is relevant as a signal-deficient strain can act as a “social cheater” in an environment where quorum sensing is required to grow^21^. Thus, we explored the ability of oxylipin-deficient strains (ΔDS or Δ*ods*R) to exploit the oxylipin signal produced by an oxylipin-proficient strain. For this purpose we co-cultured ΔDS (*PA3427*p-*lacZ*) with PAO1 and Δ*ods*R (*PA3427*p-*lacZ*) with PAO1 to measure the ability of the oxylipins produced by PAO1 bacterial cells to induce the β-gal activity in the neighbor cells of the oxylipin-deficient strains. As expected, both ΔDS (*PA3427p*-*lacZ*) and Δ*odsR* (*PA3427*p-*lacZ*) were able to sense the oxylipins produced by PAO1 and expressed a level of β-gal activity similar to that observed for PAO1 (Fig. 5a, b, respectively). These observations are in agreement with our previous results reporting that ΔDS profit from the extracellular oxylipins produced by PAO1 to complement its biofilm deficiency when they are co-cultured^16^.

### ODS is a self-regulated quorum sensing system

**Fig. 6:**
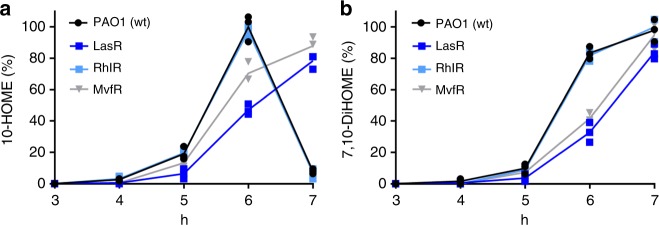
Oxylipin-dependent quorum sensing system (ODS) is self-regulated. **a** Time course of 10-HOME and **b** 7,10-DiHOME production in the supernatants of PAO1 and the transposon mutants *lasR::tn, rhlR::tn* and *mvfR::tn*. The mutants *rhlR::tn* and *mvfR::tn* showed a slower kinetics of 10-HOME and 7,10-DiHOME production. Means and s.d. are from three independent experiments. 10-HOME (10*S*)-hydroxy-(8*E*)-octadecenoic acid, 7,10-DiHOME 7*S*,10*S*-dihydroxy-(8*E*)-octadecenoic acid

*P. aeruginosa* is one of the more intensively studied model of quorum sensing systems in bacteria. In addition to the ODS system described here, this bacterium contains at least two AHL-dependent quorum sensing systems, *las* and *rhl*, and a 4-quinolone-dependent quorum sensing system, PQS^11^. These systems are interconnected forming a complex quorum sensing circuitry. In order to determine if ODS is a self-regulated system or if it is dependent of one of the above-mentioned quorum sensing systems we measured the kinetic of oxylipin production in transposon insertion mutants of the genes encoding LasR, RhlR and MvfR, which are the autoinducer receptors of *las*, *rhl* and the PQS systems, respectively. While the production of oxylipins by *rhlR::tn* showed a normal kinetic compared to PAO1, *lasR::tn and mvfR::tn* showed a slower kinetics of 10-HOME or 7,10-DiHOME production (Fig. 6a, b). However, although slower, the production of 10-HOME and 7,10-DiHOME in *lasR::tn and mvfR::tn* was still regulated in a cell density-dependent manner, demonstrating that ODS is, to a great extent, a self-regulated system.

## Discussion

**Fig. 7:**
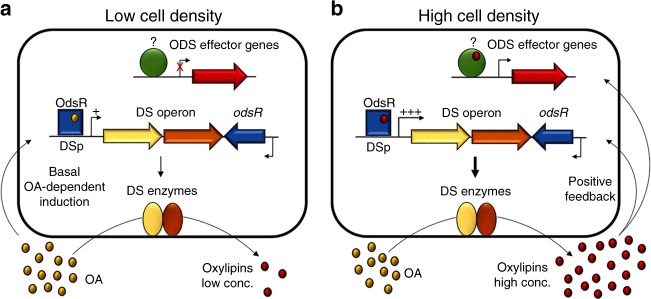
Model of the oxylipin-dependent quorum sensing system (ODS). **a** The OdsR (oxylipin-dependent diol synthase regulator) receptor (represented as a blue square) is expressed constitutively. At low cell density in the presence of oleic acid (OA), this molecule (or an unknown derivative molecule) binds OdsR inducing a basal expression of the ODS operon, shown with a single plus symbol above the DS promoter (DSp). *P. aeruginosa* grown under these conditions produces a small amount of oxylipins. **b** When the cell density increases, the oxylipins reach a threshold concentration at which they bind to OdsR. OdsR in turn induces the expression of the DS enzymes at a higher rate than that induced by OA (shown with a triple plus symbol), creating a positive regulatory feedback that further increases the extracellular concentration of oxylipins. Subsequently, the oxylipins are sensed by other as yet unidentified secondary receptor(s) (shown as a green circle) that ultimately regulate the expression of the ODS effector genes

Herein, we report a novel quorum sensing system in *P. aeruginosa* that uses oxylipins as autoinducers and regulates the expression of a gene subset in a cell density-dependent manner. The working model of ODS function is shown in Fig. 7. Based on these results, we propose a model in which the OdsR receptor is expressed constitutively. When *P. aeruginosa* encounters an environment where OA is present, this molecule directly or indirectly induces a basal expression of the diol synthase operon at low cell density. This basal induction causes a basal production of oxylipins. When the cell density increases, a certain oxylipin concentration threshold is achieved at which oxylipins bind OdsR and further activate the diol synthase operon. This positive feedback of the diol synthase operon expression causes a sudden increase in the amount of extracellular oxylipins, which in turn activates the expression of the ODS effector genes.

**Fig. 8:**
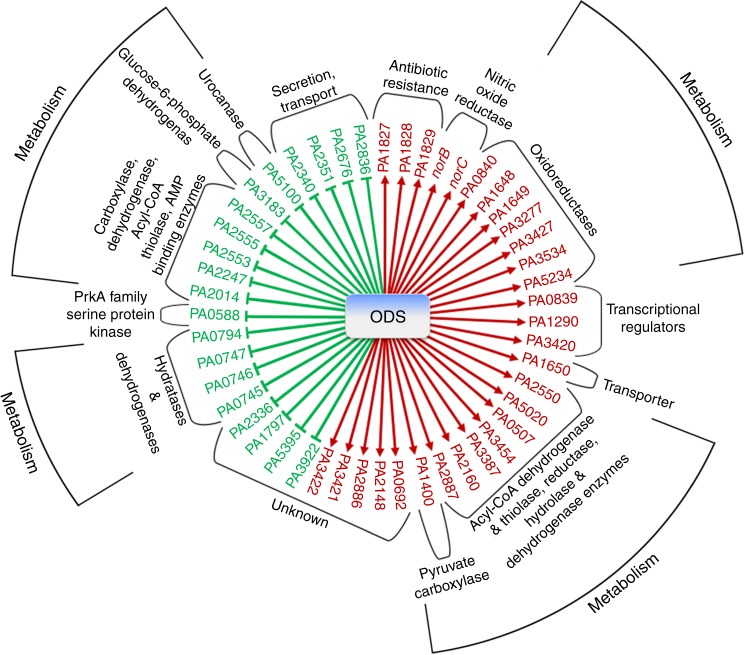
The oxylipin-dependent quorum sensing system (ODS) regulon. Categorization of genes regulated by the oleic acid (OA)-derived oxylipins in *P. aeruginosa* according to their hypothetical assigned functions in the Pseudomonas Genome Database (http://www.pseudomonas.com). Positive regulation is indicated using red arrows and negative regulation by green flat bars

The effector genes identified in this work using -omics approaches include those encoding several putative oxidoreductases, dehydrogenases, a protein kinase (*PA0588*), a putative aminoglycoside phosphotransferase (*PA1829*) and a nitric oxide reductase (*PA0523* and *PA0524*), among others (Fig. 8, Supplementary Tables 2 and 3). The roles of each of these proteins in the physiology of *P. aeruginosa* will require further extensive analysis, but their putative functions suggest that they might be involved in adaptation of *P. aeruginosa* to the host environment, resistance to oxidative stress, nitric oxide toxicity and antibiotics (Fig. 8). We have demonstrated that production and sensing of oxylipins regulates bacterial motility and thus biofilm formation, as well as virulence^16^. However, we did not identify any gene evidently related to biofilm or motility in the -omic studies. This indicates that either these processes are regulated post-transcriptionally by the oxylipins (e.g., the induction of small regulatory RNAs is a possibility) or that some of the identified hypothetical proteins might be directly involved in the regulation of motility.

The ODS system differs from other quorum sensing systems in that it uses a transcriptional regulator (OdsR) to regulate the level of oxylipin production and at least one other receptor to sense the level of oxylipins, and subsequently induces the expression of effector genes. While the biological relevance of this strategy is unknown, we speculate that the regulatory disengagement of the genes encoding the autoinducer synthetic enzymes from the regulated effector genes might confer an adaptive advantage to *P. aeruginosa*, since an OdsR mutant can still sense and respond to the oxylipins produced by neighboring cells. This does not occur with the other quorum sensing systems of *P. aeruginosa*, since the transcriptional regulator that serves as receptor of the autoinducer signal is the same that regulates the expression of the effector genes.

ODS operates as an environment-specific quorum sensing system since it requires exogenous OA as a precursor for the biosynthesis of the autoinducers. This is an interesting strategy through which *P. aeruginosa* avoids responding indiscriminately to high cell density by regulating the appropriate subset of genes required for a particular niche. OA is a fatty acid present in specific niches, such as decaying biomass and within living organisms^22,23^. This suggests that ODS plays an important role in vivo during bacterial infections. This is in agreement with our previous results indicating that *P. aeruginosa* scavenges OA from the tissues of infected *Drosophila melanogaster* to produce oxylipins, which in turn promotes bacterial virulence^16^.

The finding that *P. aeruginosa* can regulate cell density-dependent expression of the diol synthase operon even in LasR-, RhlR- and MvfR-deficient strains demonstrates that ODS is to a great extent a self-regulated quorum sensing system, and therefore abrogation of the other quorum sensing systems of *P. aeruginosa* do not annul ODS functionality. However, this does not preclude the existence of regulatory interactions of ODS with other quorum sensing systems of *P. aeruginosa*. Indeed, we found a delay in the kinetics of oxylipin production in the *lasR* and *mvfR* mutants (Fig. 5), which suggests that ODS interacts with the *las* and *mvf* quorum sensing systems at a genetic or posttranscriptional level. Due to the high complexity of the quorum sensing circuitry of *P. aeruginosa*, the determination of ODS-specific interactome with the quorum sensing network of this bacterium requires extensive investigation which is the target for future research directions.

This study provides the first evidence of a quorum sensing pathway regulated by oxylipins in bacteria. The fact that ODS has not been previously identified in the widely studied model of quorum sensing, *P. aeruginosa* highlights the importance of approaching the study of quorum sensing under conditions that better reproduce the bacterial natural environments. We believe that oxylipins could mediate cell-to-cell communication in other bacterial species, since we have identified genes homologous to *PA2077* and *PA2078* in other bacterial species, not only inside the genus *Pseudomonas*, but also in species from diverse genera, such as *Acinetobacter baumannii*, *Methylocapsa palsarum*, *Massilia eurypsychrophila and Paracoccus sanguinis* (see phylogenetic trees in Supplementary Fig. 4 and 5). Other genes encoding potential oxylipin synthases, such as lipoxygenases, di-heme cytochrome *C* peroxidases and cytochromes *P*_450_, are also widely distributed in bacteria^24–26^. Interestingly, it was recently reported that the bacterial plant pathogen *Xylella fastidiosa* produces 10-HOME and 7,10-DiHOME in vivo when inoculated in the plant model *Nicotiana tabacum*^27^. These observations suggest that production of oxylipins in bacteria is not confined to *P. aeruginosa* and thus a more general role of oxylipins in bacterial cell-to-cell communication is plausible. Yet, the discovery of ODS represents another layer of complexity in the already intricate quorum sensing circuitry of *P. aeruginosa* and a new potential target for the development of antimicrobial drugs aimed to interfere in the infection process of this important multi-drug-resistant pathogen.

## Methods

### Strains, plasmids and oligonucleotides

Strains, plasmids and oligonucleotides used in this study are described in Supplementary Table 1.

### Culture conditions

The strains were routinely grown in lysogeny broth (LB) medium at 37 °C, to which agar was added when solid medium was required. LB agar without NaCl plus 15% sucrose was used to segregate suicide plasmids from merodiploids during construction of Δ*odsR* and Δ*lasR* strains by allelic exchange (see below). When required, *P. aeruginosa* was grown in M63 media supplemented with 0.2% glucose, 0.1% casaminoacids and MgSO_4_ 1 mM (M63 complete). Antibiotics were added at the following concentrations: Ampicillin (Amp), 100 μg mL^−1^; Carbenicillin (Cb), 300 μg mL^−1^ (*P. aeruginosa*); Chloramphenicol (Cm), 25 μg mL^−1^ for *E. coli* and 200 μg mL^−1^ for *P. aeruginosa*; Kanamycin, 25 μg mL^−1^. OA 90% (Sigma) was added to cultures for oxylipin production and purification. M63-complete media were supplemented with OA 99% (Sigma) or purified oxylipins at the specified concentrations.

### Genetic constructions

Plasmid pBB-*odsR*-His was constructed by inserting *PA2076* gene and its promoter region into the pBBR1MCS plasmid^28^. The *PA2076* DNA fragment was amplified using primers *PA2076*F-*Stu*I and *PA2076*R-*Hin*dIII (Supplementary Table 1) and PAO1 chromosomal DNA as a template. The primers introduced *Stu*I and *Hin*dIII restriction sites at the extremes of the amplified fragment, which were used to insert the fragment into pBBR1MCS digested with *Hin*dIII and *Sma*I. Primer *PA2076*R-*Hin*dIII also introduced six histidine codons (coding a His-tag) at the 3’ end of the gene.

Site-directed mutagenesis of *odsR* was performed by reverse PCR. Whole plasmid pBB-*odsR*-His sequence was amplified with oligonucleotides that anneal adjacently and divergently from each other at the sequence inside *PA2076* to be mutagenized. Primer pairs used for the mutagenesis are shown in Supplementary Table 1 (primers from L91T91-F through Y201Q-R). One of the oligonucleotide pairs contained the desired base change(s) (capitalized bases, Supplementary Table 1) to change the target amino acid. The amplified fragment was then self-ligated resulting in a plasmid with identical sequence to the original except for the desired mutation inside *odsR*.

For deletion of *PA2076* from PAO1 chromosome, this gene was first amplified using primers *PA2076*F-*Eco*RI and *PA2076*R-*Hin*dIII (Supplementary Table 1), as well as PAO1 genomic DNA as template. Amplified *PA2076* fragment was *Eco*RI–*Hin*dIII digested and inserted between same sites in pEX100Tlink suicide vector^29^ to obtain pEX-*PA2076*. DNA of this plasmid was used as a template for reverse PCR amplification using primers Δ*PA2076*F-*Bam*HI and Δ*PA2076*R-*Bam*HI. Amplified fragment was *Bam*HI digested and self-ligated, resulting in plasmid pEX-Δ*PA2076*, which contains *PA2076* with an internal in-frame deletion. This suicide plasmid was used to delete *PA2076* from PAO1 chromosome by allelic replacement as previously described^28^.

Plasmid pET22b-*odsR* was ordered from GenScript. It contains an *E. coli* codon optimized *odsR* gene with a C-terminal His-Tag, which was synthesized by the vendor.

For deletion of *lasR* from PAO1 chromosome, this gene was amplified using primers *lasR*F-*Hin*dIII and *lasR*R–*Eco*RI (Supplementary Table 1), as well as PAO1 genomic DNA as template. Amplified *lasR* fragment was *Eco*RI–*Hind*III digested and inserted between same sites in pEX100Tlink suicide vector to obtain pEX-*lasR*. DNA of this plasmid was digested with *Pst*I, which cuts two times inside *lasR*, and then self-ligates, which results in plasmid pEX-Δ*lasR*, which contains *lasR* with an internal in-frame deletion. This suicide plasmid was used to delete *lasR* from PAO1 chromosome by allelic replacement as previously described^28^.

Plasmid pBB-*PA3427*p-*lacZ* contains *lacZ* gene fused to the promoter of gene *PA3427*. To construct this plasmid the *lacZ* gene from plasmid pIT2 (Manoil lab, University of Washington) was PCR-amplified using primers lacZ-5′-*Xho*I and lacZ-3′-*Bam*HI (Supplementary Table 1) that introduced *Xho*I and *Bam*HI restriction sites at respective ends of amplified DNA fragment, which was inserted into the same sites of pBBR1MCS plasmid to obtain pBB-*lacZ*. Finally, *PA3427* promoter was PCR-amplified from PAO1 chromosome using oligonucleotides *PA3427*-*Bam*HI and *PA3427*-*Xba*I and the resulting 333 bp DNA fragment was inserted between *Bam*HI/*Xba*I-digested pBB-*lacZ*, which positioned *PA3427* promoter correctly oriented in front of *lac*Z.

### Purification of 10-HOME and 7,10-DiHOME oxylipins

PAO1 was plated in LB agar and incubated overnight at 30 °C. The bacterial biomass was scraped from the plate and used to inoculate 200 mL of M63 complete supplemented with 1% oleic acid. Production of oxylipins was followed by taking 0.5 mL aliquots of the culture every 30 min, and extracting the lipids with ethyl acetate from hydrochloric acid acidified (pH = 2) supernatant aliquots (bacteria were removed by centrifugation). The lipids from each aliquot were sequentially analyzed by thin layer chromatography (TLC) until most of the OA was converted into the oxylipins. The culture was then used to purify the oxylipins. First, the culture was centrifuged at 8000 × *g* for 15 min to remove bacterial cells. The supernatant was recovered and acidified (pH = 2) with hydrochloric acid. Then, a 1 vol/vol organic extraction with ethyl acetate was carried out and the organic phase was evaporated. The dried mixture obtained was dissolved in 3 mL of ethyl acetate and used for purification of the oxylipins on an Isco Teledyne Combiflash Rf 200 with four channels with 340CF ELSD (evaporative light scattering detector). Universal RediSep solid sample loading pre-packed cartridges (5.0 g silica) were used to absorb the crude product and purified on 24 g silica RediSep Rf Gold Silica (20–40 µm spherical silica) columns using an increasing gradient of ethyl acetate (solvent B) over hexane (solvent A). Fractions collected for each detected peak were combined and evaporated, then dissolved in ethanol.

The purity of the oxylipins was analyzed by high-performance liquid chromatography/mass spectrometry analysis. Briefly, the purified 7,10 DiHOME and 10-HOME oxylipins were dissolved at 1 mg mL^−1^ in methanol (stock solution). From these solutions samples were prepared by diluting in ddH_2_O 0.1% formic acid. A 20 µL aliquot of each sample was loaded onto a Synergi Hydro-RP 80 A 250 × 2 mm C18 column (Phenomenex) using a Shimadzu Prominence System Binary Pump (Shimadzu Scientific Instruments, Inc., Columbia, MD) at a flow rate of 350 µL per min using ddH_2_O with 0.1% formic acid and acetonitrile with 0.1% formic acid for mobile phase A/B respectively. The gradient proceeded from 10–80% B over 11 min, then to 100% B at 14 min, then re-equilibrated back at initial conditions for 6 min for a total of 20 min per evaluation using the SCIEX 4000 Triple Quadrupole Mass Spectrometer (Concord, Ontario, Canada) in the electrospray ionization (ESI)-negative ion mode. Nitrogen was used as a nebulizer and curtain gas (CUR = 20). The collision gas, collision energy and temperature were set at 10 (−30 eV for 10-HOME, −34 eV for 7,10-DiHOME) and 600 °C, respectively. GS1 and GS2 gases were set at 40 and 60 respectively. Analyst 1.6.2 software controlled the liquid chromatography-tandem mass spectrometry system.

### Thin layer chromatography

TLCs were run on 60 Å silica gel plates of 20 × 10 cm and 200 µm thickness (Whatman^®^). The mobile phase solvent was a mix of hexane, ether and acetic acid (80:20:5). TLC plates were revealed with 10% phosphomolybdic acid in ethanol and dried with a hair dryer.

### Proteomics analysis

*P*. *aeruginosa* PAO1 and Δ*odsR* strains were grown in quadruple in M63 complete medium (3 mL) up to an OD600 = 1 at 30 °C and shaking (240 rpm). Each strain was then treated with 0.1 mg mL^−1^ of OA, 10-HOME or 7,10-DiHOME oxylipin or kept untreated as baseline control and incubated for additional 2 h at 30 °C and shaking (240 rpm). Bacterial cells were collected by centrifugation (10,000 rpm, 5 min) from 1 mL aliquots of each culture and frozen at −80 °C until use. Bacterial pellets were then thawed and lysed with 20 µL of NuPAGE LDS sample buffer, reduced, denatured and separated by one-dimensional polyacrylamide gel electrophoresis (1D PAGE) as previously referenced^30^. This step was carried out on an SDS Bis-Tris gel (10%, Invitrogen) as per the manufacturer’s instructions. The gels were stained, the entire lane for each sample were partitioned into 3 MW fractions and each gel plug was equilibrated in 100 mM ammonium bicarbonate (AmBc). Each gel plug was then digested with Trypsin Gold (Promega) following the manufacturer’s instruction, and peptide extracts were reconstituted in 0.1% Formic Acid/ddH_2_O at ~0.1 µg µL^−1^. Mass spectrometry runs were carried out, and the data processed, searched, filtered, grouped and quantified as previously reported in detail (Ludwig et al.^31^; under section 2.5 nLC-ESI-MS2 and Protein IDs for GeLC)^31^.

### RNA sequencing and transcriptomics analysis

*P. aeruginosa* PAO1 strain was grown in quadruplicates in M63 complete medium (3 mL) up to an OD600 = 1 at 30 °C and shaking (240 rpm). The cultures were then treated with 0.1 mg mL^−1^ of OA, 10-HOME or 7,10-DiHOME or treated with the vehicle only (ethanol) as baseline control and incubated for additional 30 min at 30 °C and shaking (240 rpm). This was done in duplicates. Bacterial cells were recovered and used to isolate total RNA using a QIAGEN RNeasy Plus Mini Kit (cat. no. 74134). Concentration and purity of RNA samples were determined by measuring the absorbance (A_260_:A_280_) using a NanoDrop 2000 spectrophotometer (ThermoFisher Scientific). Total RNA samples were then processed for the depletion of ribosomal RNA (rRNA) following the manufacturer’s user manual for Ribo-Zero rRNA removal kit for bacteria from Illumina (Document no. 15066012 V02).

RNA libraries were prepared following the Lexogen’s user manual of Sense Total RNA-seq library preparation kit (cat. nos. 009.24, 020.24, 022.24). Briefly, 10 ng of rRNA-depleted RNA was used as the starting material for the generation of libraries. The library generation started with the random hybridization of the starter/stopper heterodimer mix to the rRNA-depleted RNA. These starter/stopper heterodimers contained Illumina-compatible linker sequences. A single-tube reverse transcription and ligation reaction was then performed to extend the starter to the next hybridized heterodimer, where the newly synthesized complementary DNA insert was ligated to the stopper. The second strand synthesis was then performed to hydrolyze the RNA and then the library was converted to double-stranded DNA, which was purified using magnetic beads to adjust the library length and to ensure the complete removal of second strand synthesis reaction components. The library amplification was then performed to add the complete adaptor sequences required for cluster generation and to produce sufficient material for quality control and sequencing; i7 indices were added during this step in order to uniquely multiplex the samples for the sequencing run. The resulting RNA libraries were then purified using magnetic beads to get rid of PCR components, which could interfere with quantification and sequenced on an Illumina MiSeq instrument. Bioinformatics Analysis of RNA-seq was performed using FASTQ files from the MiSeq sequencer were uploaded to Qiagen’s GeneGlobe Data Analysis Center, where the raw sequence reads were aligned to the reference genome and transcript abundances were estimated. These transcript abundances were normalized using their Average Reference Genes’ Molecular Tags option and differential expression was calculated from these normalized values. For generating heatmaps and principal component analysis plots, ClustVis was used^32^.

### OdsR protein purification

Plasmid pET22b-*odsR* was transformed into *E.coli* BL-21 DE3 cells. A single colony was inoculated in LB medium overnight at 37 °C. A total of 5 mL of the culture was added into 500 mL autoinduction medium^33^. The cells were grown at 37 °C for 4 h, followed by overnight induction at 18 °C. The cells were harvested and cell pellets were resuspended in lysis buffer (20 mM Tris, 500 mM NaCl, 20 mM imidazole, 0.5 mM TCEP, pH 8.0) with 1 mg mL^−1^ lysozyme and 1 tablet of SIGMAFAST protease inhibitor (Sigma-Aldrich). The cell suspension was sonicated on ice and centrifuged at 15,000 rpm for 1 h. The supernatant was mixed with 1 mL Ni-NTA resin (GE Healthcare Life Sciences) and shaken for 2 h. The Ni-NTA resin was spun down and loaded onto an empty column, which was washed successively with lysis buffer and lysis buffer containing 40 mM imidazole and 60 mM imidazole. OdsR-His protein was eluted in 400 mM imidazole prepared in lysis buffer and further purified by size-exclusion chromatography (SEC) using a Superdex 200 column (16/600, GE Healthcare Life Sciences) in the SEC buffer (20 mM Tris, 150 mM NaCl, 1 mM TCEP, pH 8.0). The fractions containing Ods-His protein were pooled and concentrated to about 8 mg mL^−1^. The protein solution was aliquot and stocked at −80 °C freezer.

### Western blotting

Protein expression of *PA2076*-His (OdsR-His) or its derived single amino acid mutants was studied by western blotting using 1 µg mL^−1^ of Anti-6X His tag® antibody (HRP) conjugate (Abcam) as previously described^34^ with some modifications. Briefly, proteins were electrophoresed in 4–12% Bis-Tris Plus gels as per the manufacturer’s instructions (Invitrogen) and the resolved proteins were transferred to a polyvinylidene difluoride membrane by electroblotting using an iBlot2 apparatus (Invitrogen). The membrane was transferred to an iBind apparatus (Invitrogen), where all remaining immunoblotting steps were carried out. After last wash, the membrane was developed using SuperSignal™ West Pico (Thermo Scientific). Protein bands were imaged using ImageQuant LAS4000 imaging system (GE Healthcare Life Sciences).

### β-Galactosidase activity assay

*P. aeruginosa* strains to be assayed were grown overnight in LB agar plates, then bacterial suspensions were prepared in fresh M63 complete to OD_600_ = 0.5 with or without oxylipins or OA (0.1 mg mL^−1^). Cultures were incubated at 30 °C for the duration of the experiment, then bacterial cells were centrifuged and the supernatant discarded (to remove OA and oxylipins that interfere with the OD_600_). Bacteria were suspended in M63 medium at an OD_600_ = 1, then 250 µL of each suspension was mixed with 250 µL of Z buffer [Na_2_HPO_4_.7H_2_O (0.06 M), NaH_2_PO_4_.H_2_O (0.04 M), KCl (0.01 M), MgSO_4_ (0.001 M), b-mercaptoethanol (0.05 M), pH to 7.0], 50 µL of 0.1% SDS and 100 µl of chloroform and the mix vortexed for 20 s. The tubes were incubated at 30 °C for 5 min and the reaction started by adding 100 µl of *o*-nitrophenyl-β-D-galactoside (ONPG, 4 mg mL^−1^) and briefly vortex mixing. Reactions were incubated at 30 °C for 1 h and stopped by adding 250 µL of 1 M Na_2_CO_3_. The reaction was centrifuged for 1 min at maximum speed and the supernatant was used to measure the OD at 420 nm and at 550 nm. Finally, β-gal activity was calculated using the equation: Miller Units = 1000 × [(OD_420_ − 1.75 × OD_550_)]/(*T* × *V* × OD_600_), where OD_420_ and OD_550_ are the final reads from the reaction mixture, OD_600_ is the initial cell density of the cultures, *T* is the time of the reaction in min and *V* the volume of culture used in the assay in mL.

### Electrophoretic mobility shift assay

Binding of OdsR to the diol synthase operon (DS promoter) was assessed by EMSA using the purified OdsR-His protein. A DNA fragment comprising 200 bp immediately upstream from the *PA2078* start codon was used as a DS promoter probe. Another unrelated 150 bp DNA fragment was used as negative control. The DS promoter (15 ng) or control DNA (20 ng) probes were mixed with 0, 12, 24, 36, 48, 90 pmol of OdsR-His protein in binding buffer 1× [5× composition: 100 mM Hepes, pH 7.6, 5 mM EDTA, 50 mM (NH_4_)_2_S0_4_, 5 mM DTT, Tween 20, 1 % (w/v), 150 mM KCl] and incubated 30 min at room temperature. Each DNA-protein mix was applied in a 6% DNA retardation gel (Invitrogen Cat. No. EC6365BOX) and electrophoresed at 120 volts until the loading dye reached 1 cm from the gel edge. The gel was then stained with SYBR gold (Thermofisher), visualized and photographed.

### Co-culture experiments

Strains ΔDS (*PA3427*p-*lacZ*) and Δ*ods*R (*PA3427*p-*lacZ*) (Supplementary Table 1) were each co-cultured with PAO1 in 3 mL of M63 complete medium supplemented with OA (1 mg mL^−1^). Each pair of strains were inoculated from fresh overnight grown pre-cultures at an initial OD_600_ = 0.1 and incubated for 6 h at 30 °C. The β-gal activity was measured using 250 µL of each co-culture as described above. Strains ΔDS (*PA3427*p-*lacZ*) or Δ*ods*R (*PA3427*p-*lacZ*) alone cultured under the same conditions were used as negative controls.

### Phylogenetic tree construction

The phylogenetic trees were generated using BLAST pairwise alignments by the Neighbor Joining method. The maximum sequence difference (the maximum allowed fraction of mismatched bases in the aligned region between any pair of sequences) was set to 0.7.

### Statistical analysis

Unpaired *t*-test (two-tailed) was used to determine differences between means of varying conditions after it was determined that the variance was similar between groups. All statistical analyses were performed using GraphPad Prism 7 software.

### Reporting summary

Further information on experimental design is available in the Nature Research Reporting Summary linked to this article.

## Supplementary information

Reporting Summary

Description of Additional Supplementary Files

Supplementary Information

Supplementary Data 1

## Data Availability

The authors declare that the data supporting the findings of this study as well as new materials generated, such as plasmids and strains, are available within the article and its supplementary information files, or from the corresponding author upon request. The transcriptomic data discussed in this publication have been deposited in NCBI’s Gene Expression Omnibus^35^ and are accessible through GEO Series accession number GSE123356. The mass spectrometry proteomics data have been deposited to the ProteomeXchange Consortium via the PRIDE^36^ partner repository with the dataset identifier PXD012256.
