# Supplementary material for: Oxylipins mediate cell-to-cell communication in Pseudomonas aeruginosa
Source: Commun Biol. 2019 Feb 15;2:66. doi: 10.1038/s42003-019-0310-0 (PMC6377657; doi:10.1038/s42003-019-0310-0)
Supplement: Supplementary file 2 — Description of Additional Supplementary Files [file 42003_2019_310_MOESM2_ESM.docx]

**Description of Additional Supplementary Files**

**File Name**: Supplementary Data 1

**Description**: Raw data of all the study experiments provided in an Excel file.
