# Supplementary material for: Oxylipins mediate cell-to-cell communication in Pseudomonas aeruginosa
Source: Commun Biol. 2019 Feb 15;2:66. doi: 10.1038/s42003-019-0310-0 (PMC6377657; doi:10.1038/s42003-019-0310-0)
Supplement: Supplementary file 3 — Supplementary Information [file 42003_2019_310_MOESM3_ESM.pdf]

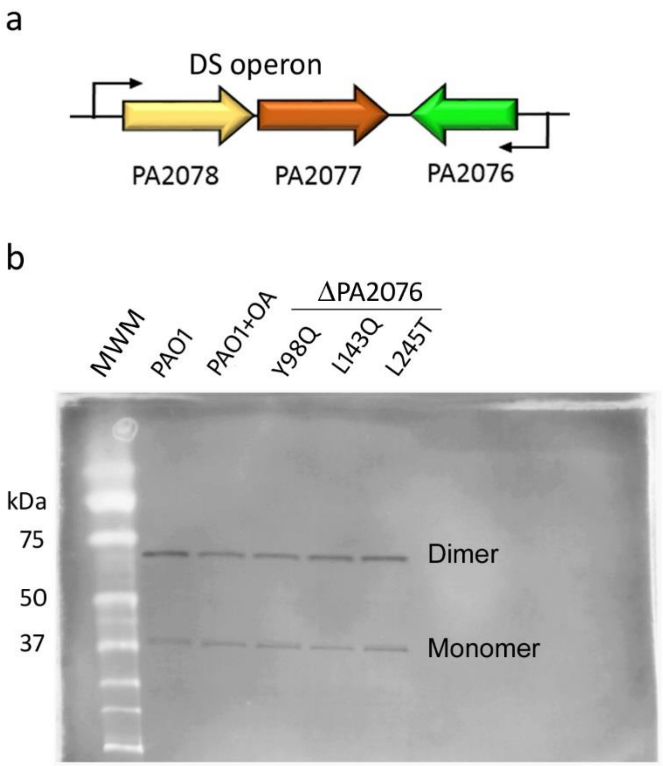

c

Strain	Plasmid	DS activity
ΔPA2076	–	–
ΔPA2076	pBB-PA2076	+
ΔPA2076	pBB-PA2076-L91T	+
ΔPA2076	pBB-PA2076-Y98Q	–
ΔPA2076	pBB-PA2076-L143Q	–
ΔPA2076	pBB-PA2076-L245T	–

Supplementary Figure 1. a Schematic representation of the DS operon including *PA2076* gene. **b** Western blot image showing the expression of PA2076 protein and its mutant variants. The first two lanes show that the expression of OdsR-His in PAO1 strain is independent of OA. The last three lanes show a normal expression of the dysfunctional PA2076 single-amino acid mutants. Both, the monomeric and dimeric forms of the proteins are visible. An anti-His tag antibody was used for detection. **c** Table showing the effect on the diol synthase activity of single amino acid mutations in the sequence of PA2076 protein. The single point mutations were evaluated in Δ*PA2076* strain as the genetic background. Δ*PA2076* transformed with pBB-*odsR-His* was used as positive control. This plasmid, which expresses the wild type protein OdsR with a histidine tail fused at the C-terminus (OdsR-His), was also used to construct the mutants. Mutations inside the hydrophobic pocket of *odsR* (Y98Q, L143Q &L245T) affected the diol synthase activity, while a distant mutation outside the pocket (L91T) had no effect.

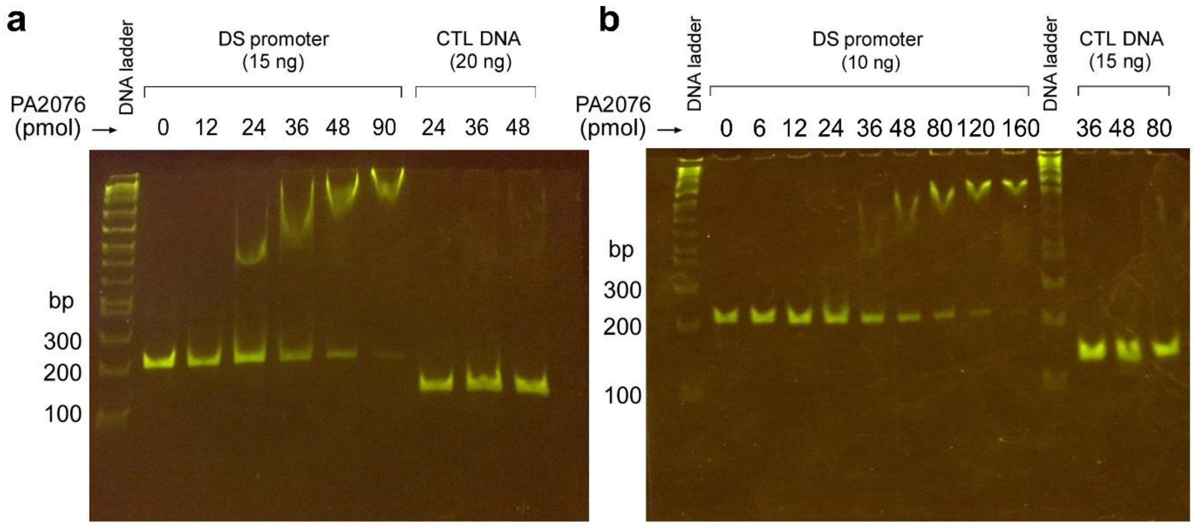

Supplementary Figure 2. EMSA assays of OdsR bound to the diol synthase operon promoter. **a** The same EMSA assay of Fig. 3c of the main article, but showing the full gel image. **b** Another independent experiment of the same EMSA assay, but using less amount of the DNA probes and a wider range of PA2076 protein concentrations.

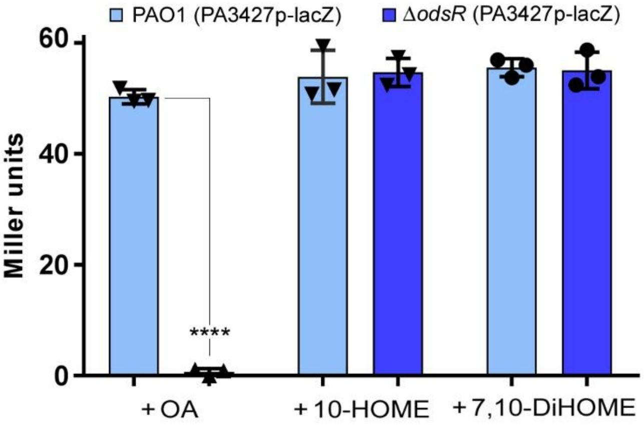

Supplementary Figure 3. 10-HOME and 7,10-DiHOME induces the expression of PA3427. Expression of β-gal activity in PAO1 (PA3427p-*lacZ*) and Δ*odsR* (PA3427p-*lacZ*) treated with OA (1 mg/ml), 10-HOME (0.1 mg/ml) or 7,10-DiHOME (0.1 mg/ml). Δ*odsR* (PA3427p-*lacZ*) expressed undetectable β-gal activity when treated with oleic acid, but the same level of β-gal activity as PAO1 (PA3427p-*lacZ*) when treated with 10-HOME or 7,10-DiHOME. Data represents the means and s.d. of three independent experiments. **** (means significantly different, unpaired two-tailed t-test, $P<0.0001$).

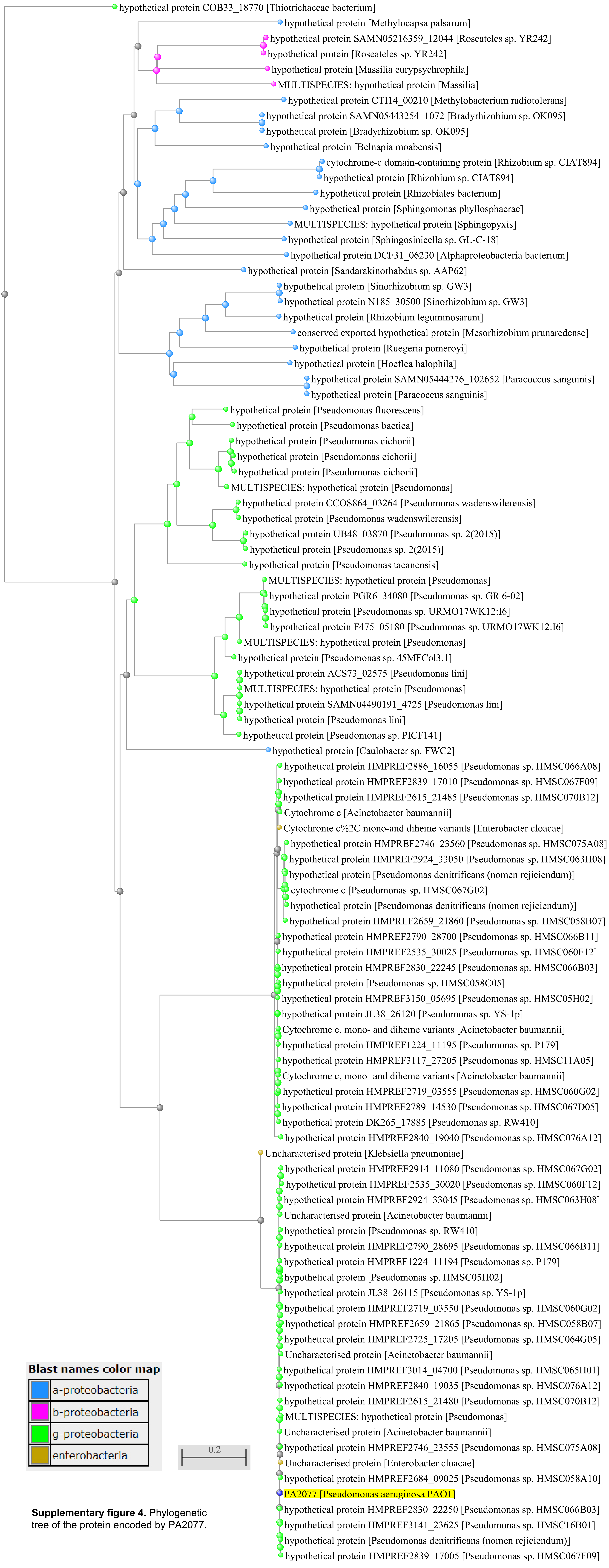

Supplementary figure 4. Phylogenetic tree of the protein encoded by PA2077.

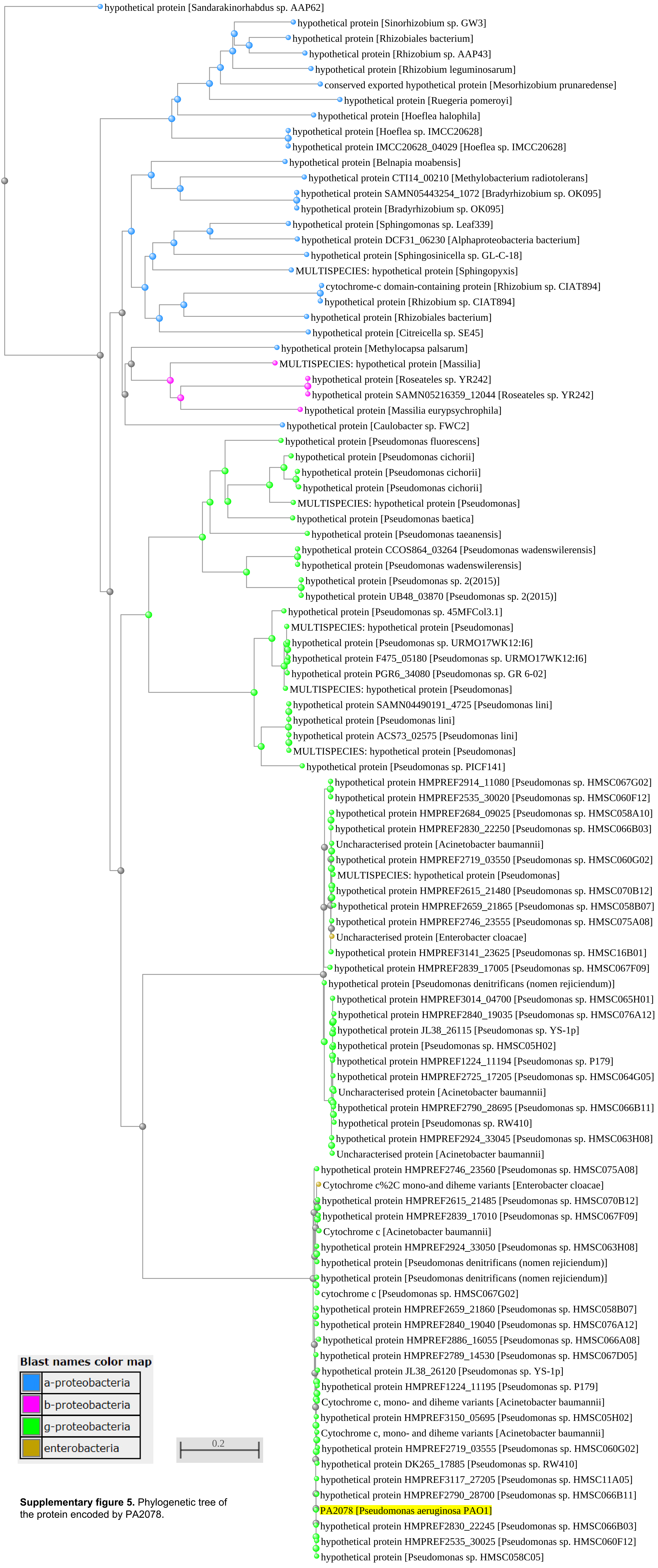

Supplementary figure 5. Phylogenetic tree of the protein encoded by PA2078.

Supplementary Table 1. Strains, plasmids and oligonucleotides used in this study.

Strains/Plasmids/ Oligonucleotides	Description or Purpose/Sequence (5'→3')	Source/ Reference
Strains:		
PAO1	*P. aeruginosa* wild type model strain.	UW*
ΔDS	PAO1 containing an in-frame deletion of genes PA2077 and PA2078, encoding the DS enzymes.	[1]
Δ*odsR*	PAO1 containing an in-frame deletion of PA2076 gene, encoding the LysR transcriptional regulator OdsR.	This study
PAO1 (pDSp-*lacZ*)	PAO1 containing plasmid pBBp-DS-*lacZ.*	This study
ΔDS (pDSp-*lacZ*)	ΔDS containing plasmid pBB-DSp-*lacZ.*	This study
ΔPA2076 (pDSp-*lacZ*)	ΔPA2076 containing plasmid pBB-DSp-*lacZ.*	This study
PW3597	PAO1 with a transposon insertion in ORF PA1430 (*lasR*).	UW*
PW6882	PAO1 with a transposon insertion in ORF PA3477 (*rhlR*).	UW*
PW2812	PAO1 with a transposon insertion in ORF PA1003 (*mvfR*).	UW*
E. coli DH5α™	Use for general cloning purposes.	Invitrogen
E. coli BL21 (DE3)	Used for OdsR-His expression.	Invitrogen
E. coli S17-1 λpir	Used as donor strain for introduction of suicide plasmids into *P. aeruginosa*.	[2]
Plasmids:		
pBBR1MCS	Conjugative multipurpose cloning vector able to replicate in *P. aeruginosa*.	[3]
pDSp-*lacZ*	pBBR1MCS containing the *lacZ* reporter gene, from *E. coli*, transcriptionally fused to the DS operon promoter.	This study
pBB-*odsR*-His	pBBR1MCS plasmid containing PA2076 (*odsR*) fused to a C-terminal His-tag and its promoter region (494 bp upstream the gene).	This study
pBB-*odsR*-L91T	pBB-*odsR*-His, where *odsR* gene contains mutation L91T.	This study
pBB-*odsR*-L245T	pBB-*odsR*-His, where *odsR* gene contains mutation L245T.	This study
pBB-*odsR*-Y98Q	pBB-*odsR*-His, where *odsR* gene contains mutation Y98Q.	This study
pBB-*odsR*-Y201Q	pBB-*odsR*-His, where *odsR* gene contains mutation Y201Q.	This study
pET22b-*odsR*	Expression vector pET22b containing an *E. coli* codon optimized *odsR* (PA2076) gene fused to a His-tag at C-terminal.	GeneScript
pBB-*PA3427*p-*lacZ*		This study
pEX100Tlink	Suicide vector used for allelic replacement in *P. aeruginosa.*	[4]
pEX-*PA2076*	pEX100Tlink containing PA2076 gene of PAO1.	This study
pEX-Δ*PA2076*	pEX-PA2076 containing an in-frame deletion in PA2076 gene plus ~550 bp to each side of the deletion. Used to create the ΔPA2076 mutant by allelic replacement.	This study
Oligonucleotides:		
PA2076F-*Stu*I	For cloning *odsR* in pBBR1MCS/atatatAGGCCTctcagcgtagcccccctg†.	This study
PA2076R-*Hind*III	For cloning *odsR* in pBBR1MCS/ atatatAAGCTTttagtggtgatggtgatgatgaccagcggctacccgtctcgcggtctcgacgaa†.	This study
PA2076F-*Eco*RI	For cloning *odsR* in pEX100Tlink/ cataGAATTCccgacacggaagcggttcac†.	This study
PA2076R-*Hind*III	For cloning *odsR* in pEX100Tlink/cataAAGCTTtgatcagtccggtgccggac†.	This study

ΔPA2076F-*Bam*HI	For internal deletion of *odsR*/cataGGATCCttcctgccgttgtcgctgac†.	This study
ΔPA2076R-*Bam*HI	For internal deletion of *odsR*/cataGGATCCCtgcgaaacgtgcagcacct†.	This study
L91T91-F	For mutagenesis of PA2076/ gcacatcggtttcagccgc.	This study
L91T91-R	For mutagenesis of PA2076/GTctggcggtcgtcgccgtc‡.	This study
L245T-F	For mutagenesis of PA2076/gaccaagttcgcctatccggg.	This study
L245T-R	For mutagenesis of PA2076/GTcgacaacggcaggaaggc‡.	This study
Y98Q-F	For mutagenesis of PA2076/aActcgatcccggctggtc‡.	This study
Y98Q-R	For mutagenesis of PA2076/Ggcggctgaaaccgatgtgc‡.	This study
Y201Q-F	For mutagenesis of PA2076/Aagccagttcatgaacgatg‡.	This study
Y201Q-R	For mutagenesis of PA2076/tGggcgggaaacagcgagtcc‡.	This study
lacZ-5'-*Xho*I	For cloning *lacZ* in pBBR1MCS/atcgCTCGAGgggatccccaccacgtggtc†.	This study
lacZ-3'-*Bam*HI	For cloning *lacZ* in pBBR1MCS/gccgGGATCCtgattacaggaggtcatac†.	This study
PA3427-*Bam*HI	For cloning PA3427 promoter in pBB- *lacZ*/ tacaGGATCCtcgggactccagggcagtag†.	This study
PA3427-*Xba*I	For cloning PA3427 promoter in pBB- *lacZ*/tacaGGATCCtcgggactccagggcagtag†.	This study

***** UW (University of Washington), Seattle, Washington (*P. aeruginosa* defined transposon library from Dr. Colin Manoil lab).
† Bases in capital letters denote the restriction site introduced by the oligonucleotide.
‡ Bases in capital letters denote the bases changed during mutagenesis.

Supplementary Table 2. List of proteins whose expression shifted five-fold or more by oleic acid, 10-HOME or 7,10-DiHOME in strains Δ*odsR* and PAO1.

		Strains and conditions*							
**Identified Proteins**	**Gene**	**Δ*odsR***	**Δ*odsR* + 7,10-DiHOME**	**ΔodsR_+ 10-HOME**	**Δ*odsR* + OA**	**PAO1**	**PAO1 + 7,10-DiHOME**	**PAO1 + 10-HOME**	**PAO1 + OA**
**Induced by 10-HOME or 7,10-DiHOME in PAO1 only:**									
oleate 10S-Dioxygenase	PA2077	0	0	0	0	0	10.612	10.726	0
oleate 7S,10S-Hydroperoxide Isomerase	PA2078	0	0	0	0	0.96326	5.306	11.799	1.8318
**Induced by 10-HOME and/or 7,10-DiHOME:**									
probable oxidoreductase	PA0840	0	9.914	29.092	4.7732	4.8163	21.224	37.541	15.57
probable oxidoreductase	PA1648	0	13.88	8.3119	2.8639	0	9.5509	13.944	8.2429
probable oxidoreductase	PA1649	0	13.88	34.287	0.95464	0	15.918	27.888	18.318
probable oxidoreductase	PA1828	3.0715	15.862	15.585	1.9093	1.9265	9.5509	11.799	19.233
putative aminoglycoside phosphotransferase	PA1829	0	14.871	17.663	0	0	24.408	30.033	7.327
probable acyl-CoA dehydrogenase	PA2550	3.0715	26.768	71.69	10.501	2.8898	50.938	82.59	44.878
hypothetical protein	PA2707	0	10.905	10.39	4.7732	1.9265	14.857	9.6534	2.7476
probable oxidoreductase	PA3277	1.0238	10.905	14.546	4.7732	0.96326	16.979	21.452	13.738
beta-ketoacyl reductase	PA3387	0	10.905	7.2729	3.8186	0	9.5509	7.5082	4.5794
probable oxidoreductase	PA3427	0	26.768	32.209	2.8639	1.9265	33.959	26.815	13.738
probable acyl-CoA thiolase	PA3454	2.0477	30.733	42.599	10.501	2.8898	39.265	42.904	25.645
probable oxidoreductase	PA3534	0	5.9484	6.234	0	0	5.306	9.6534	6.4112
probable acyl-CoA dehydrogenase	PA5020	5.1192	37.673	35.326	5.7278	1.9265	53.06	62.211	24.729
probable 3-hydroxyacyl-CoA dehydrogenase	**PA5188**	1.0238	11.897	9.3509	1.9093	0.96326	14.857	10.726	10.075
probable oxidoreductase	PA5234	0	17.845	22.858	2.8639	0	18.041	12.871	10.991
putative NAD(P) transhydrogenase, subunit alpha	**PA0195**	1.0238	5.9484	19.741	2.8639	1.9265	4.2448	19.307	6.4112
Cytochrome c oxidase, cbb3-type, CcoO subunit	**PA1556**	3.0715	0	6.234	0	0	0	8.5808	3.6635
**Inhibited by 10-HOME and/or 7,10-DiHOME:**									
putative PrkA family serine protein kinase	PA0588	32.763	15.862	1.039	21.957	33.714	25.469	0	11.906
probable enoyl-CoA hydratase/isomerase	PA0745	16.381	7.9312	1.039	14.32	17.339	15.918	3.2178	11.906
probable acyl-CoA dehydrogenase	PA0746	10.238	5.9484	0	4.7732	17.339	12.735	1.0726	4.5794
probable aldehyde dehydrogenase	PA0747	8.1907	7.9312	0	7.6371	2.8898	12.735	0	4.5794
probable aconitate hydratase	PA0794	43.001	53.535	5.195	39.14	40.457	39.265	12.871	34.803
methylcrotonyl-CoA carboxylase, beta-subunit	PA2014	29.691	24.785	0	17.183	23.118	28.653	9.6534	24.729
2-oxoisovalerate dehydrogenase (alpha subunit)	PA2247	25.596	20.819	2.078	17.183	12.522	15.918	5.363	22.897
AmbE	PA2302	16.381	19.828	1.039	27.685	35.641	12.735	2.1452	16.486
probable acyl-CoA thiolase	PA2553	45.049	21.811	2.078	22.911	43.347	35.02	11.799	13.738
probable AMP-binding enzyme	PA2555	14.334	12.888	0	15.274	20.229	12.735	0	12.822
probable AMP-binding enzyme	PA2557	4.0954	6.9398	0	6.6825	15.412	8.4897	0	4.5794
glucose-6-phosphate 1-dehydrogenase	PA3183	25.596	13.88	3.117	10.501	20.229	27.591	3.2178	5.4953
methylmalonate-semialdehyde dehydrogenase	PA3570	40.954	42.63	0	39.14	33.714	35.02	0	44.878
conserved hypothetical protein	PA3922	9.2146	10.905	0	7.6371	30.824	10.612	0	3.6635
probable binding protein component of ABC transporter	PA5096	11.262	3.9656	0	6.6825	9.6326	1.0612	0	6.4112
urocanase	PA5100	46.073	45.604	0	17.183	46.237	36.081	1.0726	27.476

* Quantitative values are normalized total MS spectra and are formatted in a red scale with deepest red corresponding to highest values. Genes highlighted in bold were not found in the transcriptomic study.

Supplementary Table 3. List of genes whose mRNA species were shifted five-fold or more by oleic acid, 10-HOME or 7,10-DiHOME in strain Δ*odsR*.

		Strain and conditions					
		**Δ*odsR* + 10-HOME**		**Δ*odsR* + 7,10-DiHOME**		**Δ*odsR* + Oleic Acid**	
**Identified Gene**	**Product**	**Fold Change***	**padj**	**Fold Change***	**padj**	**Fold Change***	**padj**
**Induced by 10-HOME and/or 7,10-DiHOME:**							
PA2148	hypothetical protein	291.0363518	0.0175779	310.981696	0.0437294	1.12114801	0.9939024
**PA2550**	acyl-CoA dehydrogenase	41.67580287	4.14E-108	8.12439848	2.678E-33	1.14187112	0.7908495
PA1400	pyruvate carboxylase	33.82846516	0.0016297	4.65401971	0.5491844	1.2171793	0.9574488
**PA1649**	short-chain dehydrogenase	27.80737387	0.0004479	10.0846487	0.0837653	-2.1136326	0.7284046
**PA1829**	putative aminoglycoside phosphotransferase	20.99029109	6.066E-62	7.99394764	1.973E-28	1.64007547	0.061515
**PA1648**	oxidoreductase	19.52508627	3.876E-41	6.06275318	1.386E-14	1.20429526	0.7675872
**PA5020**	acyl-CoA dehydrogenase	18.24466636	1.187E-58	8.14554962	3.882E-30	-1.615343	0.0668654
**PA1828**	short-chain dehydrogenase	17.73467667	1.888E-98	8.59967836	2.603E-54	1.13815013	0.7280775
PA0507	acyl-CoA dehydrogenase	14.68358923	3.933E-37	8.95826149	3.375E-24	1.12790848	0.8671616
**PA3427**	short-chain dehydrogenase	13.89868259	3.917E-24	6.334504	2.596E-11	-1.3851499	0.5793135
**PA0840**	oxidoreductase	13.02451479	6.138E-29	4.8956066	1.573E-10	-1.6693213	0.1655313
**PA3277**	short-chain dehydrogenase	10.41999313	2.517E-16	12.4250764	1.684E-19	2.18940012	0.0379833
PA1650	transporter	12.07201399	5.973E-25	4.22680356	8.163E-08	-1.0940599	0.9221831
**PA3454**	acyl-CoA thiolase	10.8784405	4.304E-50	7.60646296	1.582E-35	-1.643739	0.0224979
PA0839	transcriptional regulator	9.695057684	2.182E-28	3.02223701	3.146E-06	1.51735501	0.2338787
PA1827	short-chain dehydrogenase	9.518635955	0.0012882	7.94402199	0.0121737	1.81000363	0.6960775
**PA5234**	oxidoreductase	9.444949924	1.522E-39	4.9045934	2.973E-19	-1.5886167	0.0610518
**PA3387**	beta-ketoacyl reductase	8.460154845	1.395E-12	3.83188522	0.0001414	-1.0226174	0.9881622
PA1290	transcriptional regulator	8.329129264	1.325E-10	5.73700303	1.133E-06	1.90570974	0.2250029
PA3420	transcriptional regulator	7.740997247	1.742E-14	7.57232215	1.162E-13	-2.8725227	0.0007793
PA2886	hypothetical protein	7.457585648	1.129E-06	1.01479569	0.9964835	1.37384316	0.7636067
PA0692	hypothetical protein	6.78441856	0.0335716	-2.21751144	0.7572297	1.81031099	0.740225
PA2160	glycosyl hydrolase	6.686721418	8.414E-07	1.39954764	0.8363738	1.87657968	0.3266506
PA2887	citronellol catabolism dehydrogenase	6.235702824	0.0043339	1.99011534	0.6974364	1.20439447	0.9276971
**PA3534**	oxidoreductase	6.10944333	3.734E-24	1.79694746	0.0264812	1.00692309	0.997067
PA0524	nitric-oxide reductase subunit B	5.009518675	1.669E-05	4.06603811	0.0009042	2.24676639	0.1064654
PA0523	nitric-oxide reductase subunit C	4.947589837	2.937E-07	5.12340972	5.205E-07	1.2725066	0.7640553
PA3422	hypothetical protein	3.131326093	3.08E-06	9.92773103	5.693E-21	-1.772791	0.020458
PA3421	hypothetical protein	2.421177721	3.744E-06	20.9633378	0.0437294	-2.1162469	0.0001169
**Inhibited by 10-HOME and/or 7,10-DiHOME:**							
PA5395	hypothetical protein	-6.16105677	0.001206	-1.8455982	0.269115	-1.9974241	0.212418
PA2863	lipase chaperone	-7.0199635	0.000187	1.40189462	0.503555	-1.4141334	0.494265
PA2676	type II secretion system protein	-8.51737488	0.000384	-1.6090638	0.417513	1.26067029	0.69203
PA2340	binding-protein-dependent maltose/mannitol transport protein	-10.4557662	0.001273	-2.57802193	0.184489	-1.1131177	0.880189
PA1797	hypothetical protein	-10.8768087	3.49E-10	-1.54678898	0.23974	-1.3154923	0.459623
PA2836	secretion protein	-13.0990421	0.003438	-2.17893683	0.361022	-1.3506178	0.72387
PA5541	dihydroorotase	-24.5442316	0.00026	-3.38721583	0.143328	-1.0833823	0.923041
PA2336	hypothetical protein	-35.9081479	0.003139	-1.74717664	0.597896	1.17199522	0.880288
PA2351	ABC transporter permease	-111.646199	0.004079	-5.21948664	0.22071	3.67626486	0.318355
PA4862	ABC transporter ATP-binding protein	-143.518517	0.003794	1.62952067	0.726922	-1.2421766	0.877123

*Fold change values were calculated with respect to Δ*odsR* grown in the absence of oxylipins or OA. Values in red indicate > five-fold induction, while values in green indicate > five-fold inhibition. Highlighted genes in bold were found in the proteomic study also.

Supplementary References

1. Martínez, E. & Campos-Gómez, J. Oxylipins produced by Pseudomonas aeruginosa promote biofilm formation and virulence. *Nat. Commun.* **7**, 13823 (2016).
2. de Lorenzo, V. & Timmis, K. N. Analysis and construction of stable phenotypes in gram-negative bacteria with Tn5- and Tn10-derived minitransposons. *Methods Enzymol.* **235**, 386–405 (1994).
3. Kovach, M. E., Phillips, R. W., Elzer, P. H., Roop, R. M. & Peterson, K. M. pBBR1MCS: a broad host-range cloning vector. *BioTechniques* **16**, 800–802 (1994).
4. Quénée, L., Lamotte, D. & Polack, B. Combined sacB-based negative selection and cre-lox antibiotic marker recycling for efficient gene deletion in pseudomonas aeruginosa. *BioTechniques* **38**, 63–67 (2005).
